# Analysis of Refractive Index Sensing Properties of a Hybrid Hollow Cylindrical Tetramer Array

**DOI:** 10.3390/nano15020118

**Published:** 2025-01-15

**Authors:** Meng Wang, Paerhatijiang Tuersun, Aibibula Abudula, Lan Jiang, Dibo Xu

**Affiliations:** 1 Xinjiang Key Laboratory for Luminescence Minerals and Optical Functional Materials, School of Physics and Electronic Engineering, Xinjiang Normal University, Urumqi 830054, China; wangmeng091999@163.com (M.W.); jianglan021999@163.com (L.J.); xu13597881988@163.com (D.X.); 2 School of Medical Technology, Xinjiang Hetian College, Hetian 848011, China

**Keywords:** hybrid nanoarrays, bound states in the continuum, refractive index sensing, finite difference time domain method, optical sensing

## Abstract

In recent years, metal surface plasmon resonance sensors and dielectric guided-mode resonance sensors have attracted the attention of researchers. Metal sensors are sensitive to environmental disturbances but have high optical losses, while dielectric sensors have low losses but limited sensitivity. To overcome these limitations, hybrid resonance sensors that combine the advantages of metal and dielectric were proposed to achieve a high sensitivity and a high *Q* factor at the same time. In this paper, a hybrid hollow cylindrical tetramer array was designed, and the effects of the hole radius, external radius, height, period, incidence angle, and polarization angle of the hollow cylindrical tetramer array on the refractive index sensing properties were quantitatively analyzed using the finite difference time domain method. It is found that the position of the resonance peaks can be freely tuned in the visible and near-infrared regions, and a sensitivity of up to 542.8 nm/RIU can be achieved, with a *Q* factor of 1495.1 and a figure of merit of 1103.3 RIU^−1^. The hybrid metal–dielectric nanostructured array provides a possibility for the realization of high-performance sensing devices.

## 1. Introduction

In the field of optics, the way in which to control and constrain the propagation of light is a hot issue that has attracted much attention for a long time. For example, optical fibers, optical resonators, etc., are typical optical components that constrain light. However, they cannot completely constrain the incident light, and there will inevitably be some leakage. The discovery of bound states in the continuum (BICs) broke this bottleneck, and it is a unique optical phenomenon. Although BICs exist in the continuum, it does not radiate any energy outwards and is a perfect bound state [1].

The concept of BICs was first proposed by Von Neumann and Wigner in quantum mechanics in 1929 [2]. These states correspond to special resonances located within the radiated continuum while there is no energy decay [3]. Since then, BICs have been widely studied in the water wave [4], sound wave [5], electromagnetic wave [6], and other fields. In 1985, Friedrich and Wintgen [7] proposed a more efficient way to construct BICs, which were generated by the interference of the resonance states between different channels, and BICs were later called Friedrich–Wintgen BIC. In 1999–2003, Fan et al. [8,9] used the time-domain coupling mode theory to explain the BIC phenomenon in photonic crystals. In 2008, Marinica et al. [6] extended the BIC concept further to optical systems, and since then, it has become a new way to enhance the interaction between light and matter. In 2011, Plotnik et al. [10] firstly realized the construction of the symmetric protected BIC with the help of the optical waveguide array structure, and broke the structural symmetry by adding a refractive index gradient, so that the BIC leaked into the continuum and generated quasi-BICs (qBICs). In recent years, optical BICs have been successfully realized in photonic crystals [11], metasurfaces [12], and surface plasmons [13]. Optical BICs have a wide range of applications, including lasers [14], sensors [15], fiber structures [16], etc.

Optical sensors have gradually become the focus of researchers, and they have applications in various research fields [17,18,19,20,21]; for example, environmental monitoring [22], chemical characterization [23], biosensing [24], etc. Optical sensors can be roughly divided into two types according to material—metal-based surface plasmon resonance sensors [25,26] and dielectric-based guided-mode resonance sensors [27,28]. Compared to dielectric sensors, metal surface plasmon resonance sensors usually have a large near-field enhancement and a high sensitivity [25]. However, due to the large internal ohmic damping losses and radiation losses, the metal surface plasmon resonance mode in the visible and near-infrared spectral regions typically have a wide spectral linewidth; therefore, the *Q* factor is low and the figure of merit is small [29,30,31]. On the other hand, guided-mode resonance sensors can produce a high *Q* resonance by employing dielectric materials. All-dielectric metasurface structures generally have low losses, are compatible with complementary metal-oxide semiconductors, and have a high *Q* factor enabled by Fano resonance [32]. Since the non-radiative loss of dielectric materials is lower than that of metals, the increased possibility of Fano resonance, the increased resonance intensity, and the reduced spectral width are beneficial for sensor applications [33,34]. For most optical BICs based on dielectric structures [35], the inverse square dependence relationship between the asymmetric parameters and the *Q* factor has been extensively studied and clearly clarified [36,37], but their sensitivity and figure of merit—two important indicators to evaluate sensing performance—are still worthy of better exploration [20]. To overcome these limitations, hybrid resonance sensors that combine the advantages of metal and dielectric were proposed to achieve a high sensitivity and a high *Q* factor.

In this paper, a hybrid metal–dielectric hollow cylindrical tetramer array is proposed. The effects of size parameters, period, incident light, and other factors on the refractive index sensing properties are quantitatively analyzed using the finite difference time domain (FDTD) method. According to the simulated data, the refractive index sensitivity (RIS), *Q* factor, and figure of merit (*FOM*) in different resonance modes were calculated, and the physical mechanism behind the variation was analyzed. The hybrid nanostructure array provides a possibility to realize high-performance sensing devices.

## 2. Model and Methods

In this work, the refractive index sensing properties of the hybrid hollow cylindrical tetramer array are numerically simulated using FDTD, and the reflectance spectrum, refractive index sensitivity, *Q* factor, and figure of merit are calculated.

**Figure 1:**
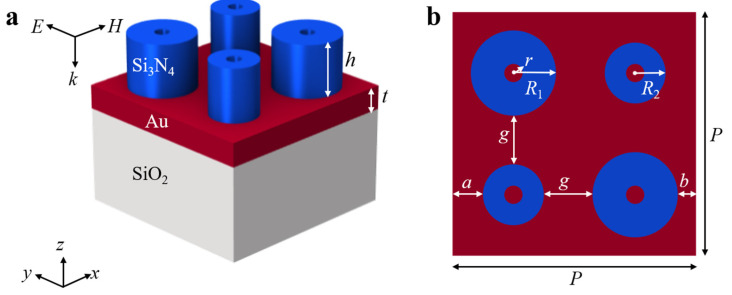
The unit structure, (**a**) Three-dimensional diagram and (**b**) *xoy* plane diagram of the hybrid hollow cylindrical tetrameric array.

Figure 1 shows the geometric model (Figure 1a) and *xoy* plane diagram (Figure 1b) of the hybrid hollow cylindrical tetramer array unit structure. The structure consists of a SiO_2_ substrate, a Au layer, and a Si_3_N_4_ hollow cylindrical tetramer. We assume that the top layer (Si_3_N_4_) height *h* is 225 nm, the hole radius *r* is 40 nm, the thickness *t* of the Au layer is 100 nm, the bottom SiO_2_ substrate is semi-infinitely thick, and the period *P* of each structural unit is set to 800 nm in the *x* and *y* directions. In addition, the two Si_3_N_4_ hollow cylinders on each diagonal have the same size (*R*_1_ represents the radius of the large hollow cylinder and *R*_2_ represents the radius of the small hollow cylinder). In the simulation, only the TE polarization of the electric field parallel to the *y* direction is considered. The incident beam (plane wave) is directed along the negative *z*-axis. Periodic boundary conditions are set in the *x* and *y* directions, while perfectly matched layer boundary conditions are applied in the *z* direction to absorb the outgoing scattered light. The grid spacing in the *x*, *y*, and *z* directions is set to 5 nm. All simulations were carried out using Ansys Lumerical FDTD 2023 R2.1. The refractive index of the Si_3_N_4_ and SiO_2_ substrates is set as 2 and 1.45, respectively, and the refractive index of the surrounding environment is set as 1.3. The refractive index of Au is taken from the published paper written by Johnson and Christy [38].

Bulk refractive index sensitivity (*S*_bulk_), a widely used sensing indicator that indicates the ability to structurally sense changes in the environmental refractive index of the entire region, is defined as follows:

(1) $$S_{\mathrm{bulk}}=\frac{\Delta \lambda_{\mathrm{res}}}{\Delta n},$$

where Δ*n* is the change in the environmental refractive index and Δ*λ*_res_ is the change in resonance wavelength with Δ*n*.

For optical sensors, the *Q* factor is often used to describe the performance stability of the sensor. It reflects the relationship between the energy storage and energy consumption of optical systems. Specifically, a high *Q* factor means that the system has a lower energy loss and a higher energy storage capacity. The *Q* factor is defined as follows:

(2) $$Q=\frac{\lambda_{\mathrm{res}}}{\mathrm{FWHM}},$$

where *λ*_res_ is the resonance wavelength and FWHM is the full width at half maximum of the resonance.

In addition, *FOM* is used to evaluate the comprehensive resolving power of optical sensors. It can be defined as follows [39]:

(3) $$FOM=\frac{S_{\mathrm{bulk}}}{\mathrm{FWHM}}$$

## 3. Results and Discussion

The refractive index sensing properties of nanoarrays are significantly affected by the size, period, light source, and surrounding environment. In order to better understand the variation in refractive index sensing properties with these influencing factors, we select the hybrid hollow cylindrical tetramer array as the research object, and quantitatively analyze the effects of hole radius, small hollow cylinder external radius, cylinder height, period, incident light, and surrounding environment on the refractive index sensing properties. Except for the analysis of the influence of the surrounding environment on the refractive index sensing properties, the refractive index of the surrounding environment is taken as 1.3; Δ*n* is taken as 0.02 to calculate the refractive index sensitivity.

### 3.1. Effect of Size on Refractive Index Sensing Properties

In order to reveal the variation in the refractive index sensing properties of the hybrid hollow cylindrical tetramer array with the size parameters, the reflectance spectra of the hybrid hollow cylindrical tetramer array with the change in the hole radius, the external radius of the small hollow cylinder, and the height of the cylinder were quantitatively analyzed, and the corresponding bulk refractive index sensitivity (*S*_bulk_), *Q* factor, and figure of merit (*FOM*) were calculated.

#### 3.1.1. Effect of Hole Radius

**Figure 2:**
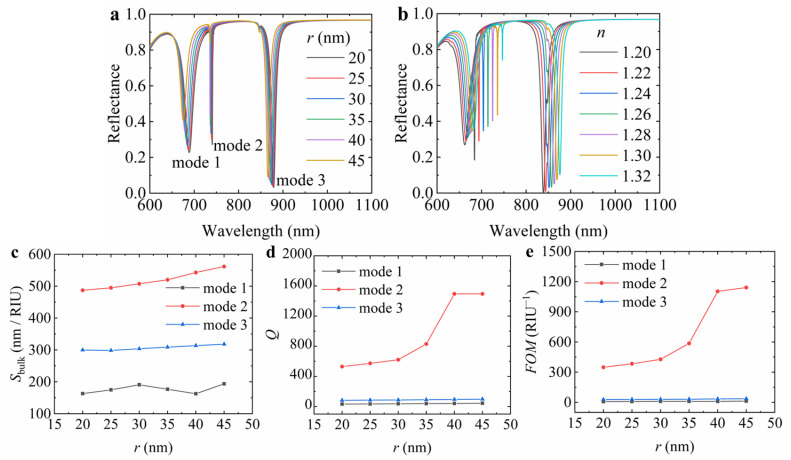
The reflectance spectrum of the hybrid hollow cylindrical tetramer array varies with (**a**) hole radius *r* and (**b**) environmental refractive index *n* and their corresponding values of (**c**) *S*_bulk_, (**d**) *Q* factor, and (**e**) *FOM*. In the simulation, the external radius *R*_1_ of the large hollow cylinder is 140 nm, the external radius *R*_2_ of the small hollow cylinder is 100 nm, the period *P* is 800 nm, the height *h* of Si_3_N_4_ is 225 nm, and the thickness *t* of the Au layer is 100 nm.

Firstly, without changing the surrounding environment, we calculated the reflectance spectra of the hybrid hollow cylindrical tetramer array with a hole radius of 20 nm, 25 nm, 30 nm, 35 nm, 40 nm, and 45 nm, respectively. Note that the hole radii of large and small cylinders are the same. The results are shown in Figure 2a. It can be found that there are three resonance valleys in the reflectance spectra. With the increase in hole radius, the resonance wavelengths at the three modes blueshift, and the resonance intensities are weakened gradually. The resonance intensity of mode 2 is smaller compared to the other resonance modes. Compared to the FWHM of mode 1, the FWHM of mode 3 is narrower and has a larger resonance intensity. To further verify the bulk refractive index sensitivity of the structure, we selected hybrid hollow cylinder tetramer arrays with a hole radius of 40 nm; the refractive index of the surrounding environment is changed between 1.20 and 1.32 with a step of 0.02; the corresponding shift in the reflectance spectra can be observed in Figure 2b. With the increase in the refractive index, all the resonance wavelengths at the three modes redshift, and the resonance intensities are weakened gradually. This is because when the refractive index of the surrounding environment increases, the effective refractive index of the light in the structure also increases. It causes the propagation speed of the light to slow down and the interaction of the light with the structure to enhance, thus causing the resonance wavelength to redshift. By comparing Figure 2c–e, it is found that the *S*_bulk_, *Q* factor, and *FOM* at mode 2 are larger. With the increase in hole radius, the *S*_bulk_, *Q* factor, and *FOM* increase gradually. When the hole radius increases to 45 nm, the resonance at mode 2 is weakened, as shown in Figure 2a. Therefore, for the following analysis, the hole radius is set to 40 nm, the corresponding bulk refractive index sensitivity is 542.8 nm/RIU, the *Q* factor is 1495.1, and the figure of merit is 1103.3 RIU^−1^.

**Figure 3:**
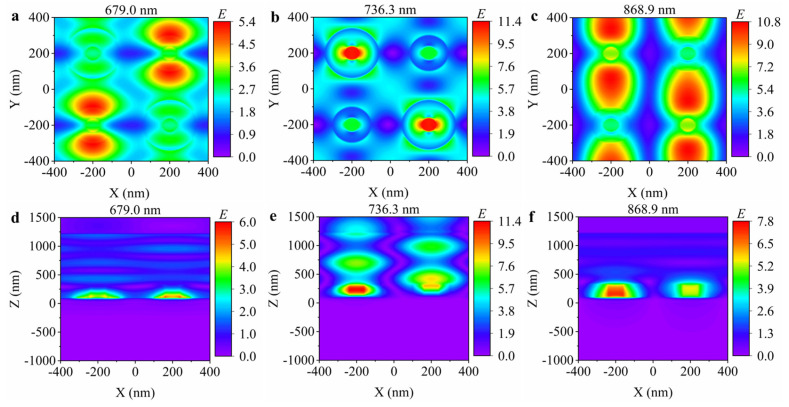
Electric field distributions in the (**a**–**c**) *xoy* plane and (**d**–**f**) *xoz* plane at the resonance wavelengths of 679.0 nm, 736.3 nm, and 868.9 nm, respectively.

Figure 3a–c show the electric field distribution in the *xoy* plane at the three resonance wavelengths (679.0 nm, 736.3 nm, and 868.9 nm). According to the properties of the electric field distribution, mode 1 is the surface plasmon resonance (SPR) mode that occurs at the interface between metal and dielectric, mode 2 is the dielectric-dominated qBIC (d-qBIC) mode, and mode 3 is the metal-dominated qBIC (m-qBIC) mode. Both the d-qBIC and m-qBIC mode are generated by breaking the C_2_ symmetry in the *x* and *y* directions, and their field distributions show odd symmetry in the in-plane direction. In addition, we simulate the electric field distribution in the *xoz* plane of the hybrid hollow cylindrical tetramer array, as shown in Figure 3d−f. Most of the electric field of the SPR is confined to the interface between the gold film and the Si_3_N_4_ layer, and the electric field of the d-qBIC is concentrated in the resonance region and has no obvious attenuation in the substrate. The field distribution indicates the excellent bulk sensing ability of the hybrid hollow cylindrical tetramer nanostructure array. In addition, the electric field of the m-qBIC is localized near the surface of the gold film and partially extends into the Si_3_N_4_ layer.

#### 3.1.2. Effect of External Radius of the Small Hollow Cylinder

**Figure 4:**
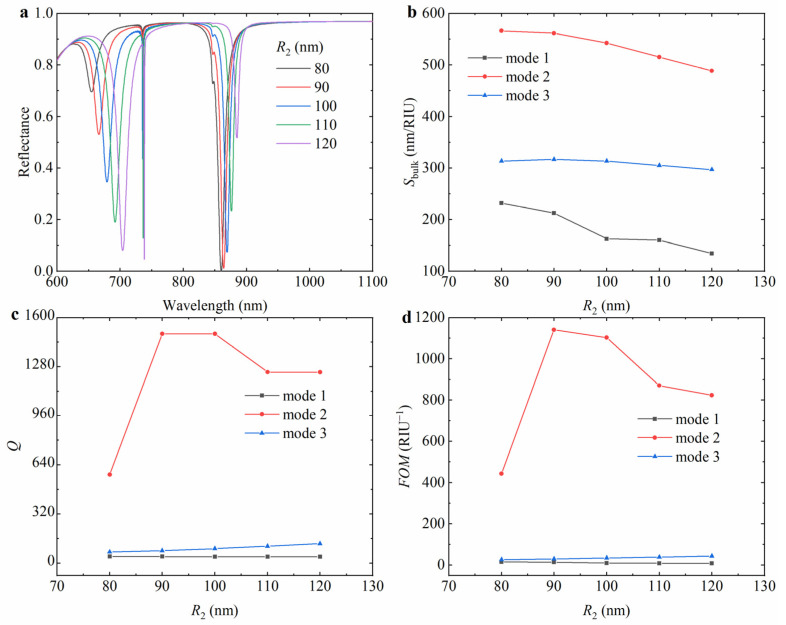
The variation in (**a**) reflectance spectrum with the external radius *R*_2_ of the small hollow cylinder and the corresponding (**b**) *S*_bulk_, (**c**) *Q* factor, and (**d**) *FOM* of the hybrid hollow cylindrical tetramer array. In the simulation, the external radius *R*_1_ of the large hollow cylinder is 140 nm, the hole radius *r* of the hollow cylinder is 40 nm, the period *P* is 800 nm, the height *h* of Si_3_N_4_ is 225 nm, the thickness *t* of the Au layer is 100 nm, and the environmental refractive index *n* is 1.3.

To quantitatively analyze the effect of different small hollow cylinder external radii on the reflectance spectra of the hybrid nanostructure array, the reflectance spectra of the hybrid hollow cylindrical tetramer array with external radii of 80 nm, 90 nm, 100 nm, 110 nm, and 120 nm were calculated, as shown in Figure 4a. The resonance wavelengths redshift as the external radius increases, and the resonance intensities of mode 1 and mode 2 are gradually enhanced, while the resonance intensity of mode 3 is gradually weakened. When the external radius of the small hollow cylinder increases, the scattering process of light in the structure is enhanced, the propagation path of light becomes longer, and the resonance wavelength shifts towards the longer wavelength direction. Combined with Figure 4b–d, it is not difficult to see that mode 2 has a better performance than mode 1 and mode 3. As the external radius increases, the *S*_bulk_ of mode 1 decreases from 232.2 nm/RIU to 134.7 nm/RIU, the *S*_bulk_ of mode 2 decreases from 566.4 nm/RIU to 488.9 nm/RIU, and the *S*_bulk_ of mode *3* increases from 313.8 nm/RIU to 316.6 nm/RIU first and then decreases to 297.0 nm/RIU. At the same time, the *Q* factor and *FOM* of mode 2 increase first and then decrease, while the *Q* factor and *FOM* of mode 3 show a slight increasing trend.

#### 3.1.3. Effect of Cylinder Height

**Figure 5:**
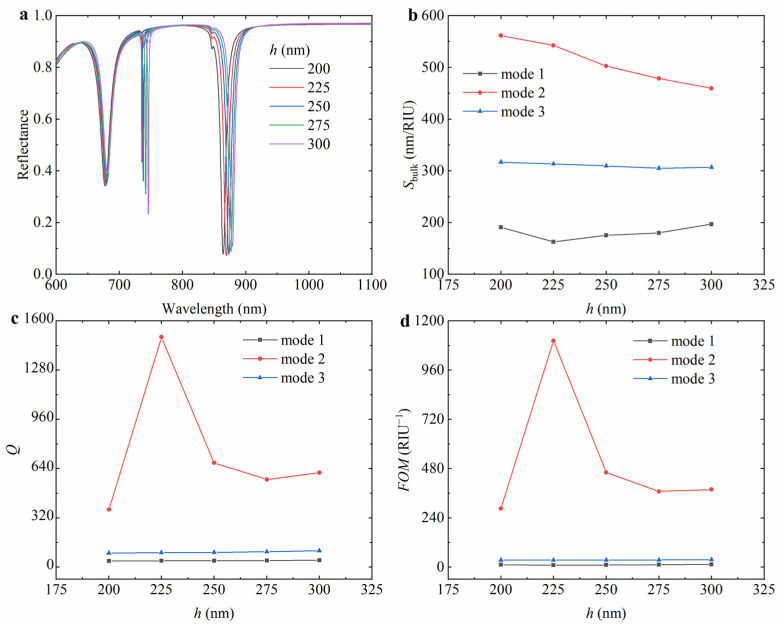
The variation in (**a**) reflectance spectrum with the cylindrical height *h* and the corresponding (**b**) *S*_bulk_, (**c**) *Q* factor, and (**d**) *FOM* of the hybrid hollow cylindrical tetramer array. In the simulation, the external radius *R*_1_ of the large hollow cylinder is 140 nm, the external radius *R*_2_ of the small hollow cylinder is 100 nm, the hole radius *r* of the hollow cylinder is 40 nm, the period *P* is 800 nm, the thickness *t* of the Au layer is 100 nm, and the environmental refractive index *n* is 1.3.

To quantitatively analyze the effect of different cylinder heights on the reflectance spectra of the hybrid nanostructure array, the reflectance spectra of the hybrid hollow cylindrical tetramer array with cylinder heights of 200 nm, 225 nm, 250 nm, 275 nm, and 300 nm were calculated, as shown in Figure 5a. With the increase in the cylinder height, all the resonance wavelengths redshift, the resonance intensity of mode 1 is gradually weakened, the resonance intensity of mode 2 is gradually enhanced, and the resonance intensity of mode 3 is slightly enhanced first and then gradually weakened. This is because the increase in the height of the cylinder makes the propagation path of light in the dielectric longer, increasing the time and intensity of the interaction between the light and the structure, resulting in the resonance wavelength redshifting. As shown in Figure 5b–d, the *S*_bulk_ of mode 1 decreases from 191.0 nm/RIU to 162.7 nm/RIU first and then increases to 196.9 nm/RIU with the increase in cylindrical height. The *S*_bulk_ of mode 2 decreases with the increase in cylindrical height, and the corresponding *Q* factor increases from 374.0 to 1495.1 first and then decreases to 614.0, and the *FOM* increases from 285.6 RIU^−1^ to 1103.3 RIU^−1^ first and then decreases to 378.3 RIU^−1^. With the increase in cylindrical height, the *S*_bulk_ of mode 3 decreases from 316.9 nm/RIU to 305.1 nm/RIU first and then increases to 306.8 nm/RIU, and the corresponding *Q* factor and *FOM* have no obvious change.

### 3.2. Effect of Period on Refractive Index Sensing Properties

**Figure 6:**
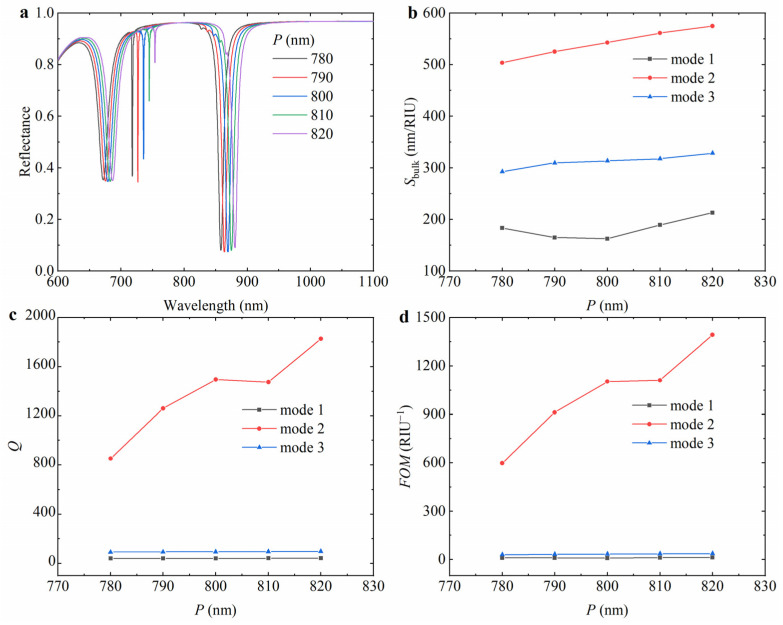
The variation in (**a**) reflectance spectrum with the period *P* and the corresponding (**b**) *S*_bulk_, (**c**) *Q* factor, and (**d**) *FOM* of the hybrid hollow cylindrical tetramer array. In the simulation, the external radius *R*_1_ of the large hollow cylinder is 140 nm, the external radius *R*_2_ of the small hollow cylinder is 100 nm, the hole radius *r* of the hollow cylinder is 40 nm, the height *h* of Si_3_N_4_ is 225 nm, the thickness *t* of the Au layer is 100 nm, and the environmental refractive index *n* is 1.3.

To quantitatively analyze the effect of different periods on the reflectance spectrum of the hybrid nanostructure array, the reflectance spectra of the hybrid hollow cylindrical tetramer array with periods of 780 nm, 790 nm, 800 nm, 810 nm, and 820 nm were calculated, as shown in Figure 6a. The resonance wavelengths redshift with the increase in the period, and the resonance intensity of mode 2 is first strengthened and then weakened. When the period increases, the distance between the unit structures becomes wider, which will change the light scattering and interference of the structure, so that the resonance wavelength shifts to the direction of the longer wavelength. As shown in Figure 6b–d, with the increase in the period, the *S*_bulk_ of mode 1 first decreases from 183.7 nm/RIU to 162.7 nm/RIU and then increases to 213.1 nm/RIU, the corresponding *Q* factor increases gradually, and the *FOM* decreases first and then increases. The *S*_bulk_ of mode 2 increases from 503.4 nm/RIU to 574.9 nm/RIU, the corresponding *Q* factor increases from 851.5 to 1825.3, and the *FOM* increases from 597.2 RIU^−1^ to 1392.0 RIU^−1^. The *S*_bulk_ of mode 3 increases from 292.6 nm/RIU to 328.9 nm/RIU, the corresponding *Q* factor increases from 90.2 to 97.6, and the *FOM* increases from 30.8 RIU^−1^ to 36.4 RIU^−1^.

### 3.3. Effect of Incident Light on Refractive Index Sensing Properties

**Figure 7:**
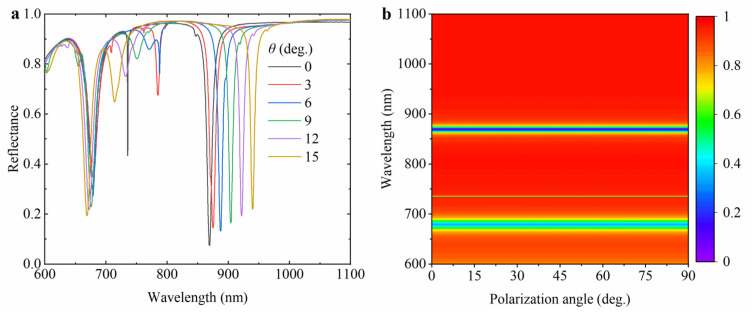
The variation in reflectance spectrum of the hybrid hollow cylindrical tetramer array with (**a**) incidence angle *θ* and (**b**) polarization angle of the incident light.

To further reveal the influence of incident light on the hybrid hollow cylindrical tetramer array, the reflectance spectrum of the hybrid hollow cylindrical tetramer array with different incidence angles *θ* (the angle between the direction of incident light and the normal direction.) was simulated and analyzed with the change in wavelength, as shown in Figure 7a. With the increase in the incidence angle, the resonance wavelength of mode 3 redshifts, and the resonance intensity of mode 2 is gradually weakened. This is because when the incidence angle increases, the coupling effect of light between structures will be enhanced, resulting in an increase in the optical path difference, which shifts the resonance wavelength of the reflectance spectra in the direction of the longer wavelength. In our previous analysis, we found that mode 2 performed better compared to mode 1 and mode 3. However, with the increase in the incidence angle, the resonance intensity of mode 2 gradually weakens and may even disappear. Therefore, the incidence angle cannot be too large in practical applications. Finally, we investigated the effect of polarization angle on the reflectance spectrum, which is often a concern in potential applications. In fact, one motivation for designing a two-dimensional asymmetric structure array is our desire for the polarization-insensitive performance of the structure. Figure 7b shows, in the reflectance spectra, that when the polarization angle of the normal incident light (with respect to the *x*-axis) is changed, the position and spectral width of the reflectance resonance valleys hardly change, which proves the polarization insensitivity of the structure.

## 4. Conclusions

In this paper, a hybrid hollow cylindrical tetrameric array is designed, and the mechanism of its refractive index sensing properties is investigated. The dependence of resonance properties on the structure size, period, incident light, and refractive index of the surrounding environment is analyzed. In addition, we demonstrated that hybrid metal–dielectric nanostructure arrays can be used for refractive index sensing applications. In this work, we obtained a sensing system with a low loss and a narrow resonance by combining metal surface plasmon resonance and dielectric guided-mode resonance, thereby improving sensitivity and obtaining a high *Q* factor and *FOM*. For the designed hybrid hollow cylindrical tetramer array, the sensitivity is 542.8 nm/RIU, the *Q* factor is 1495.1, and the *FOM* is 1103.3 RIU^−1^. By changing the size or period of the structure, the nanostructure array can obtain good wavelength tunability. A high sensitivity and a low loss make hybrid metal–dielectric nanostructure arrays advantageous in sensor applications. With advances in current nanofabrication methods, it is possible to design and fabricate such hybrid devices on a large scale.

## Data Availability

The original contributions presented in this study are included in the article; further inquiries can be directed to the corresponding authors.
